# Quality of Life of Brazilian Vegetarians Measured by the WHOQOL-BREF: Influence of Type of Diet, Motivation and Sociodemographic Data

**DOI:** 10.3390/nu13082648

**Published:** 2021-07-30

**Authors:** Shila Minari Hargreaves, Eduardo Yoshio Nakano, Heesup Han, António Raposo, Antonio Ariza-Montes, Alejandro Vega-Muñoz, Renata Puppin Zandonadi

**Affiliations:** 1 Department of Nutrition, Faculty of Health Sciences, Campus Darcy Ribeiro, University of Brasilia (UnB), Asa Norte, Brasilia 70910-900, DF, Brazil; shilaminari@gmail.com (S.M.H.); renatapz@unb.br (R.P.Z.); 2 Department of Statistics, University of Brasilia, Brasilia 70910-900, DF, Brazil; eynakano@gmail.com; 3 College of Hospitality and Tourism Management, Sejong University, 98 Gunja-Dong, Gwanjin-gu, Seoul 143-747, Korea; 4 CBIOS (Research Center for Biosciences and Health Technologies), Campo Grande 376, Universidade Lusófona de Humanidades e Tecnologias, 1749-024 Lisboa, Portugal; 5 Social Matters Research Group, Universidad Loyola Andalucía, C/Escritor Castilla Aguayo, 4 14004 Córdoba, Spain; ariza@uloyola.es; 6 Public Policy Observatory, Universidad Autónoma de Chile, Santiago 7500912, Chile; alejandro.vega@uautonoma.cl

**Keywords:** vegetarian diet, vegetarianism, quality of life, WHOQOL-BREF

## Abstract

This study aimed to evaluate the general quality of life (QoL) of Brazilian vegetarians. A cross-sectional study was conducted with Brazilian vegetarian adults (18 years old and above). Individuals were recruited to participate in a nationwide online survey that comprised the WHOQOL-BREF as well as sociodemographic and characterization questions related to vegetarianism. The WHOQOL-BREF is composed of 24 items which are divided into four domains (domain 1: physical health; domain 2: psychological well-being; domain 3: social relationships; and domain 4: environment), plus two general items which were analyzed separately, totaling 26 items. The answers from the questionnaire were converted into scores with a 0–100 scale range, with separate analyses for each domain. Results were compared among groups based on the different characteristics of the vegetarian population. A total of 4375 individuals completed the survey. General average score results were 74.67 (domain 1), 66.71 (domain 2), 63.66 (domain 3) and 65.76 (domain 4). Vegans showed better scores when compared to the other vegetarians, except in domain four, where the statistical difference was observed only for semi-vegetarians (lower score). Individuals adopting a vegetarian diet for longer (>1 year) showed better results for domains one and two, with no difference for the other domains. Having close people also adopting a vegetarian diet positively influenced the results for all domains. On the other hand, it was not possible to distinguish any clear influence of the motivation for adopting a vegetarian diet on the scores’ results. Adopting a vegetarian diet does not have detrimental effects on one’s QoL. In fact, the more plant-based the diet, and the longer it was adopted, the better the results were.

## 1. Introduction

According to the World Health Organization (WHO), quality of life (QoL) refers to how an individual feels about their goals, expectations, patterns, and fears, in the cultural and value system in which he/she is inserted [1]. It is a concept that involves physical, psychological, social and spiritual dimensions, as well as the perception of well-being [2,3].

WHOQOL-BREF is among the most common tools used to measure QoL. It is used to evaluate global QoL, being related to individuals’ satisfaction with their life [4]. It comprises 26 items, with 24 items divided into four domains (physical health, psychological well-being, social relationships, and environment), plus two general items not included in the domains. In Brazil, a translated version has already been tested, showing good psychometric performance and being considered a good option for evaluating overall QoL [5]. Other studies have already used the WHOQOL-BREF in Brazil to evaluate QoL in the general population [6], as well as in other specific population groups, such as the elderly [7,8,9,10], individuals with depression [11,12], pregnant women [13], and students [14,15,16].

It has already been demonstrated that adopting a specific dietary pattern can influence one’s QoL. By following a more restrictive or alternative diet, individuals can face difficulties finding options to eat, and might feel excluded when participating in social situations, which are often food centered [17]. In this way, their QoL could be negatively affected. On the other hand, adopting specific diets can have a positive impact on QoL. After following a diet as a treatment for a specific condition, individuals who have diseases (such as celiac disease, for example) might see an improvement in symptoms and, therefore, see improvements in their QoL [18].

Vegetarianism is a broad terminology that comprises non-conventional diets in which animal products are totally or partially excluded, and it can be classified into four main types of diet: semi-vegetarian or flexitarian (allows meat intake no more than once per week); pesco-vegetarian or pescatarian (excludes all meats except fish); ovolacto-vegetarian (excludes all meats but includes eggs and dairy) and strict vegetarian (excludes all animal products) [19]. Since vegetarianism is an alternative dietary pattern compared to a traditional western diet (followed by the majority of the western population), vegetarians may face difficulties regarding food options, or social events and situations [20,21]—which could result in worse QoL. On the other hand, the choice of adopting a vegetarian diet is mainly related to ethical/moral reasons, spiritual and religious factors, health improvement, and a wish to reduce the environmental impact caused by food production. Vegetarians could, therefore, feel good about their choice of doing something that benefits them, and protects animals and the planet. In addition, they may feel a deep sense of being part of a bigger cause [22], which could potentially result in a positive impact on QoL.

Vegetarians’ quality of life has been assessed in studies in other countries using different tools and focusing on specific population subgroups. These studies used both general and specific questionnaires to evaluate QoL [23,24,25,26,27]. The specific questionnaires were not related to vegetarianism but to other features of the studied population, such as the Obesity and Weight-Loss Quality of Life (OWQOL) and the Weight-Related Symptoms (WRSM) questionnaires [27]; the Quality of Life related to Dietary Changes questionnaire [26]; and the Food Acceptability Questionnaire (FAQ) [25].

In Brazil, no studies evaluating the general QoL of vegetarians were found. Our research group developed and applied a specific tool to evaluate the QoL of vegetarians (VEGQOL). Results showed that vegetarians have a good quality of life, with better results among vegans [28]. Despite the advantages of using a specific tool to evaluate QoL among vegetarians in Brazil, a global study using a general tool is also relevant for the comprehension of how a vegetarian diet might affect QoL, as well as to allow comparisons with other population groups evaluated with the same method. Therefore, this study aimed at evaluating the QoL of Brazilian vegetarians using the WHOQOL-BREF.

## 2. Materials and Methods

### 2.1. Study Design

This cross-sectional study carried out with Brazilian vegetarians used an instrument divided into two parts to evaluate their quality of life: (I) sociodemographic data, which included gender, age, housing location, average income, educational level and self-reported weight and height (used to calculate body mass index [BMI]). Questions related to vegetarianism were also included in order to characterize our study population: (a) type of vegetarian diet (vegan, ovolactovegetarian, pesco-vegetarian and semi-vegetarian); (b) time of adopting the diet (less than one year, between one and five years, or more than five years/always); (c) main motivation for adopting a vegetarian diet (ethical/moral, personal health, religion/beliefs, environmental aspects, aversion/intolerance, others); and (d) close people also adopting the diet (yes/no); and (II) WHOQOL-BREF [29], used to evaluate general aspects of the quality of life of vegetarians.

### 2.2. WHOQOL-BREF

The WHOQOL-BREF is composed of 26 items, of which 24 are divided into four domains: (I) physical health (seven items): pain/discomfort, energy/fatigue, sleep and rest, dependence on medication, mobility, activities of daily living, and working capacity; (II) psychological well-being (six items): positive feelings, negative feelings, self-esteem, thinking/learning/memory/concentration, body image, and spirituality/religion/personal beliefs; (III) social relationships (three items): personal relations, sex, and practical social support; and (IV) environment domain (eight items): financial resources, information/skills, recreation/leisure, home environment, access to health/social care, physical safety/security, physical environment, and transport. The two extra items are related to general quality of life perception and satisfaction with health [29].

### 2.3. Subjects

Brazilian vegetarian adults (aged 18 and above) were recruited for the study. Individuals were invited to participate via email, social networks, and messaging. The Brazilian Vegetarian Society and other support groups/influencers in the area helped to publicize the research and reach more people. The sample size described by Hair et al. [30] was used considering an error (e) of 3% and a level of significance (α) of 5%. Since no official data describing the size and distribution of the Brazilian vegetarian population were available, data from MapaVeg [31] (an independent national project that collects data about the vegetarian population in Brazil) were used. The minimum estimated sample, based on MapaVeg data (*n* = 29,282), would be 1030 participants.

### 2.4. Procedures

Data were collected using the SurveyMonkey^®^ tool in 2019. Volunteers received the invitation by email or message, or were reached by a social media post to which a weblink was attached. By clicking on it, they would be redirected to a Consent Form, and then to the questionnaire.

### 2.5. Data Analysis

Results from WHOQOL-BREF were analyzed separately (and not as a single final score), as recommended by the World Health Organization [29]. For each item, possible answers are given on a five-point Likert scale. Results were classified according to the direct or inverse score (from one to five) for each answer. Scores for each domain item were added, and the result was divided by the number of items of the corresponding domain. The final result was then converted to a 0–100 scale ([domain score − 1] × 100/4]. Results from the two general items were used as indicators of overall QoL, described separately [1,32].

The categorical variables were described as frequencies and percentages, and the quantitative variables as mean and standard variation or standard error. Independent Student t-test, Anova with Tukey’s post-hoc tests and Ancova with Bonferroni post-hoc tests were used to examine differences in each variable’s scores. The Chi-square test was used to compare categorical variables. The level of statistical significance was set at 5% (*p* < 0.05). The statistical software IBM SPSS Statistics for Windows (IBM Corp, Armonk, NY, USA) was used for the analyses.

## 3. Results

### 3.1. Sociodemographic Data

From the initial sample of 5401 that accessed the survey, 81% (*n* = 4375) of the participants completed the WHOQL-BREF. Therefore, this was considered our final sample. Based on the established criteria, the sample was considered representative of the Brazilian vegetarian population, as the number of participants was higher than the minimum sample size estimated at 1030 individuals, and the distribution across all the country’s 27 Federative Units was similar to the one from MapaVeg (Supplementary file Table S1).

Body mass index (BMI) was calculated based on the self-reported weight and height, and was then classified into nutritional status categories [33]. Results from sociodemographic data, nutritional status and sample characteristics are described in Table 1.

### 3.2. Quality of Life

Results from WHOQOL-BREF were analyzed separately by each of the four domains. Considering our total sample and a 100-point scale, average results were: 74.67 (domain 1), 66.71 (domain 2), 63.66 (domain 3) and 65.76 (domain 4). The two general items were also analyzed considering the total sample. For items 1 (“How would you rate your quality of life?”) and 2 (“How satisfied are you with your health?”), the average scores were 78.92 (SD = 18.08) and 73.01 (SD = 22.60), respectively.

Analyses were conducted considering the different characteristics of vegetarians. Regarding the types of diet, results were better for vegans across all domains. For domain 1 (physical), the more restricted (in terms of animal foods) a diet was, the better the score. The same trend was observed in domains 2 (psychological well-being) and 3 (social relationships), although no statistically significant difference was observed between vegetarians and pesco-vegetarians. For domain 4 (environment), only semi-vegetarians differed statistically from the other groups, showing a lower score.

The length of time following the diet also influenced the QoL, but only for domains 1 and 2. People who have been following a vegetarian the diet for less than one year showed lower scores compared to the others. The motivation for adopting the diet did not seem to influence results. Despite some minor differences in scores, it was not possible to observe any trend. Finally, having close people also adopting a vegetarian diet positively influenced the scores in all domains. The results are presented in Table 2.

Despite the heterogeneous distribution according to the sociodemographic characteristics among individuals following different types of diet (Supplementary file Table S2), the QoL results obtained for each type of diet after the corrected analyses were essentially equal to those found prior to correction (Supplementary file Table S3).

## 4. Discussion

The WHOQOL-BREF has been widely used to evaluate QoL in different populations all over the word, as it is considered a valid cross-cultural tool capable of reflecting one’s QoL based on its four domains [29]. This is the first study that evaluated general QoL of Brazilian vegetarians using the WHOQOL-BREF.

So far, no studies have analyzed general QoL among vegetarians considering different variables and subcategories of this population. In general, our results showed that vegans, those who have followed a vegetarian diet for longer (above one year), and those who had close people also adopting the diet had better results. Previous results published about Brazilian vegetarians, using the specific tool developed to evaluate the QoL of vegetarians (VEGQOL), showed similar results: the more plant-based a diet, the better the score. On a 100-point scale, QoL average score was 79.21 ± 10.66 for vegans, 73.13 ± 11.58 for vegetarians, 69.55 ± 12.50 for pesco-vegetarians and 64.38 ± 12.84 for semi-vegetarians. Scores were also better for those who had been following the diet for longer: 75.82 ± 12.71 and 73.84 ± 12.05 for those adopting a vegetarian diet for more than five years and more than one year, respectively, compared to 70.21 ± 12.32 for those who had adopted the diet less than one year before. The participants who have people close to them adopting a vegetarian diet also had better results: 74.57 ± 12.21 compared to 70.75 ± 12.89 for those who did not [28].

A study conducted in German-speaking countries with long-distance runners (158 vegetarians and 123 omnivores) using the WHOQOL-BREF found high scores for both groups, with no statistical differences. The authors concluded that a vegetarian diet did not have any detrimental effect on the QoL of runners [24]. In the United States, a 22-week health program was implemented at a workplace where volunteers should follow a vegan diet. Results showed improvements on participants’ QoL, measured with the Short Form-36 (SF-36) [25]. QoL was also measured in type 2 diabetes patients who followed a vegetarian diet for 24 weeks. Compared to controls (following a standard diet for diabetes treatment), those who followed a vegetarian diet showed more QoL improvements, measured with the Obesity and Weight-Loss Quality of Life (OWQOL) questionnaire [27]. Our study did not have a group composed of omnivores to enable a similar comparison. However, we found converging results since our data showed that the more restricted a diet was (in terms of animal product intake), the better the QoL scores were in almost all the evaluated aspects.

A cross-sectional study conducted in Germany compared the QoL of vegans and vegetarians using the WHOQOL-BREF questionnaire. Results from the survey showed that vegans had higher scores on three (physiological, psychological, and social) of the four domains of the WHOQOL-BREF (and no difference for the environmental domain) [23]. Similarly, we found better results for vegans for the same three domains than all other types of vegetarians, and no difference in the fourth domain, except for semi-vegetarians (who had a lower score).

The WHOQOL-BREF has already been used to evaluate QoL among the general Brazilian population. A study conducted in the South of Brazil with a total sample of 751 individuals found lower scores compared to those found in this study, especially for the physical domain (58.9 ± 10.5 versus 74.67 ± 15.96 in our study) and for the environment domain (59.9 ± 14.9 versus 65.76 ± 14.92). The average score for the psychological domain was also lower but was closer to that found in our study (65.9 ± 10.8 versus 66.71 ± 16.90). In contrast, results were better for the social domain score (76.2 ± 18.8 versus 63.66 ± 20.17) [6]. Another study conducted in Belo Horizonte/Brazil with 930 individuals also found lower scores for the physical domain (63.0 ± 18.1 versus 74.67 ± 15.96 in our study) and the environmental domain (52.4 ± 15.5 versus 65.76 ± 14.92). The average score for the psychological domain was similar to that found in our study (66.5 ± 16.3 versus 66.71 ± 16.90), and was slightly better for the social domain (68.2 ± 20.4 versus 63.66 ± 20.17) [34].

The lower results found in our study for the social domain score might be related to the fact that following a different dietary pattern may negatively affect social interactions (which are often centered around food), since individuals might feel excluded [19]. Vegetarianism may be considered not only a dietary choice but a social identity, and the fear of being stigmatized may lead vegetarians to make exceptions and eat meat [35], which demonstrates how relevant it is for some individuals to be socially accepted. People who adopt a vegetarian diet might also suffer from discrimination. Negative behaviors towards vegetarians (especially vegans) are known as “vegaphobia”. It is hypothesized that vegetarians are rejected because their presence would be a reminder that eating meat is not necessary and, therefore, not justifiable [36]. However, surprisingly, when analyses were made considering different types of vegetarian diets in our study, vegans scored better in the social domain than vegetarians and pesco-vegetarians (intermediate scores), and the worst result was found in semi-vegetarians, which would be the closest group to omnivores. Possibly, a sense of connection with other people who share the same life philosophy could positively influence the social domain. A qualitative study conducted with young vegan women showed that becoming vegan brought to them a sense of connection with the vegan community, with a positive influence on social relationships [22].

On the other hand, the higher average score related especially to the physical domain might reflect better health in vegetarians. An umbrella review of meta-analyses published in 2020 showed that vegetarians had a reduced risk of negative health outcomes, such as diabetes, cancer, and ischemic heart disease [37]. Moreover, vegetarians have lower overweight and obesity rates and better diet quality than nonvegetarians [38,39]. A better health overall could positively influence the physical health domain score in the WHOQOL-BREF, since it is composed of aspects related to discomfort, energy, sleep and rest, dependence on medication, mobility, daily activities, and working capacity, all of which could be impaired in people with poorer health. It has already been demonstrated in a cross-sectional study with people with different diseases and conditions that ill individuals had lower scores in the WHOQOL-BREF physical domain when compared to healthier individuals [40].

Our study found a link between better results and diets with higher levels of plant-based foods for three of the four domains. This might be related to the fact that vegetarianism is usually adopted due to a personal choice, mostly related to reducing animal suffering and environmental impact, as well as for religious/spiritual reasons and personal health. Therefore, adopting a vegetarian diet might increase a sense of positiveness in people related to doing something good for others and themselves. In this case, the more restricted a diet is in terms of animal product intake, the higher this sense of achievement would be, which could explain the better sense of overall QoL.

The lack of a difference (except for semi-vegetarians) observed in the environment domain score might show that there are no differences among groups regarding access to a healthy environment. Having limited access to a wide variety of foods could negatively affect the environment domain scores. In Brazil, access to fresh fruits and vegetables may be influenced by family income, with individuals in the lower-income categories making more choices based on food prices rather than their preferences. However, meat represents the most expensive item among all household food expenses [41], which is in line with the worse results found for semi-vegetarians (the only ones who still eat all types of meat). In this sense, following a more plant-based diet would positively affect the environment domain, at least in the economic field. In our study, average income distribution was very similar among the different types of diets (Supplementary file Table S2), except for pesco-vegetarians, who had a slightly higher proportion of individuals with an income above five minimum wages. Therefore, access to a variety of foods was probably similar among groups.

Having close people also adopting the diet was shown to have an important influence on QoL. This result is in line with the findings of Rosenfeld and Tomiyama [35], who showed that the main reason for vegetarians to make exceptions and eat meat is social pressure from friends, colleagues, and family. Avoiding discomfort, feeling the need to cause a good impression, or fearing to be rejected were reasons given by vegetarians to justify their occasional meat consumption. Therefore, having close people also adopting a vegetarian diet might make it easier to avoid uncomfortable situations and potential social rejections, positively reflecting on one’s QoL.

The time needed for adaptation to a new diet might explain the better results found among those following a vegetarian diet for longer (more than one year) for domains 1 and 2. A short period might not be enough to generate perceivable differences in health parameters, which could positively affect domain 1. The potential difficulties faced when adopting a new diet pattern might also somehow influence a person’s psychological well-being. On the other hand, following a diet for longer might lead to more positive feelings and improve aspects connected to self-esteem and spirituality, all of which are connected to domain 2. Domains 3 and 4, on the other hand, might not be influenced by time since they refer to relations with other people and environmental aspects, which can be considered external factors, and will depend on the context in which an individual is inserted, which will not necessarily vary with time.

This study has some limitations. First of all, despite its large size, a convenience sample was used, making it harder to generalize the results. On the other hand, since the proportion of vegetarians in Brazil is low, using a convenience sample made it possible to reach individuals at a national level and to do subgroup analyses with different subcategories, which had not yet been done in other studies. In addition, our sample was composed mainly of female subjects (62.1%), which could also be seen as a limitation. However, other studies with vegetarians have shown the same pattern, with over 60% of the participants being women [42,43]. Meat consumption is often associated with masculinity [44], and men are more resistant to giving up meat [45]. Therefore, we believe that a higher female proportion most likely represents the real gender distribution among vegetarians.

Our sample was composed mainly of younger individuals, with a very low proportion of participants above 60 years old (2.5%). Lower engagement in online surveys could help explain these results. In Brazil, only 45.0% of individuals in the age category of 60 years old and above used the internet in 2019, compared to a range of 74.2% up to 92.7% in the other age categories [46]. However, it is also possible that our results represent, at least partially, the real age distribution of vegetarians in Brazil. In the EPIC-Oxford study (*n* = 65,429), when comparing meat-eaters and different categories of vegetarian diets, the more plant-based the diet was, the lower the average age [42]. Our analyses also revealed that a higher proportion of vegans and vegetarians were below the age category of 40 compared to pesco- and semi-vegetarians (Supplementary file Table S2). In this sense, it is plausible to infer that younger people are more prone to adopting a vegetarian diet, which would contribute to a higher representation of younger individuals in our sample.

## 5. Conclusions

To date, this is the first study to evaluate the overall QoL of a nationwide vegetarian population using a non-specific tool and considering different subcategories and features of vegetarians. The results obtained in this study provide a better understanding of the impact of a vegetarian diet on one’s general QoL. Based on these results, it is possible to affirm that adopting a vegetarian diet does not have detrimental effects on QoL. In fact, the more plant-based the diet and the longer it has been followed, the better the results.

## Figures and Tables

**Table 1:** Characteristics of the participants.

Characteristic	Category	Respondents (*n* = 4375)	
		Number	Percentage
Gender	Male	1657	37.9%
	Female	2718	62.1%
Age	18–24	1166	26.7%
	25–29	735	16.8%
	30–39	848	19.4%
	40–49	1040	23.8%
	50–59	476	10.9%
	60 or more	110	2.5%
Housing location	Capital or metropolitan area	2951	67.5%
	Urban area (other cities)	1296	29.6%
	Rural area	128	2.9%
Average income ^(1)^	Less than two minimum wages	648	14.8%
	Between two and five minimum wages	1308	29.9%
	Between five and ten minimum wages	1202	27.5%
	Between ten and twenty minimum wages	701	16.0%
	Above twenty minimum wages	250	5.7%
	Not informed	266	6.1%
Educational level	No education	0	0.0%
	Elementary School, incomplete	2	0.0%
	Elementary School, complete	13	0.3%
	High School, incomplete	45	1.0%
	High School, complete	483	11.0%
	University level, incomplete	1090	24.9%
	University level, complete	2742	62.7%
BMI ^(2)^	<18.5 kg/m^2^	233	5.3%
	18.5 to 24.9 kg/m^2^	2844	65.0%
	24.9 kg/m^2^ to 29.9 kg/m^2^	923	21.1%
	>29.9 kg/m^2^	338	7.7%
	Not informed	37	0.8%
Type of diet	Vegan	1391	31.8%
	Vegetarian	2098	48.0%
	Pesco-vegetarian	330	7.5%
	Semi-vegetarian	556	12.7%
Time adopting	Less than 1 year	1040	23.8%
the diet	Between 1 and 5 years	1931	44.1%
	More than 5 years	1404	32.1%
Main motivation	Ethic/moral	2661	60.8%
	Personal health	495	11.3%
	Religion/beliefs	209	4.8%
	Environmental impact	541	12.4%
	Aversion/intolerance	158	3.6%
	Others	311	7.1%
Close people also	No	1152	26.3%
adopting the diet	Yes	3223	73.7%

^(1)^ One minimal wage is equivalent to R$1045.00 or US$232.74 (in 2020); ^(2)^ Source: [33].

**Table 2:** Mean and standard deviation (SD) for WHOQOL-BREF domains, according to the sample characteristics.

Characteristic	WHOQOL-BREF Domains			
	Physical Health	Psychological Well-Being	Social Relationships	Environment
	Mean (SD)	Mean (SD)	Mean (SD)	Mean (SD)
All subjects	74.67 (15.96)	66.71 (16.90)	63.66 (20.17)	65.76 (14.92)
Gender				
Male	75.75 (15.17) ^a^	65.87 (17.31) ^a^	63.30 (20.64) ^a^	64.29 (15.13) ^a^
Female	74.01 (16.39) ^b^	67.22 (16.64) ^b^	63.89 (19.87) ^a^	66.65 (14.72) ^b^
*p* ^(2)^	0.000	0.011	0.349	0.000
Type of diet				
Vegan	78.60 (14.45) ^a^	69.86 (16.01) ^a^	65.01 (20.33) ^a^	67.29 (14.46) ^a^
Vegetarian	74.12 (15.81) ^b^	66.03 (17.23) ^b^	64.33 (19.63) ^b^	65.53 (15.04) ^a^
Pesco-vegetarian	71.76 (16.97) ^c^	65.29 (16.00) ^b^	61.94 (19.38) ^b^	66.44 (14.58) ^a^
Semi-vegetarian	68.61 (16.99) ^d^	62.25 (16.98) ^c^	58.8 (21.47) ^c^	61.99 (15.16) ^b^
*p* ^(1)^	0.000	0.000	0.000	0.000
Time adopting diet				
Less than 1 year	73.19 (16.28) ^a^	64.95 (16.78) ^a^	62.49 (20.12) ^a^	65.36 (14.86) ^a^
Between 1 and 5 years	75.07 (15.60) ^b^	66.69 (16.82) ^b^	64.06 (20.04) ^a^	65.63 (14.79) ^a^
More than 5 years	75.21(16.16) ^b^	68.03 (17.00) ^b^	63.99 (20.36) ^a^	66.22 (15.14) ^a^
*p* ^(1)^	0.003	0.000	0.100	0.334
Main motivation				
Ethic/moral	74.78 (15.94) ^a^	66.31 (17.05) ^a,b^	63.36 (20.37)) ^a^	65.91 (15.09) ^a,b^
Personal health	74.70 (16.95) ^a^	68.68 (15.80) ^b^	63.87 (19.19) ^a^	66.53 (14.73) ^b^
Religion/beliefs	76.04 (15.05) ^a^	69.32 (15.33) ^b^	64.35 (19.34) ^a^	65.06 (14.46) ^a,b^
Environmental impact	75.67 (13.93) ^a^	67.19 (16.25) ^a,b^	65.33 (19.51) ^a^	65.71(14.31) ^a,b^
Aversion/intolerance	69.67 (17.90) ^b^	63.90 (18.53) ^a^	62.18 (21.89) ^a^	62.94 (17.03) ^a^
Others	72.99 (16.89) ^a,b^	65.86 (18.20) ^a,b^	63.34 (20.73) ^a^	65.21 (13.88) ^a,b^
*p* ^(1)^	0.000	0.002	0.356	0.149
Close people also adopting the diet				
No	72.91 (16.94) ^a^	64.16 (17.97) ^a^	59.43 (20.50) ^a^	63.96 (15.39) ^a^
Yes	75.29 (15.55) ^b^	67.62 (16.42) ^b^	65.17 (19.83) ^b^	66.40 (14.70) ^b^
*p* ^(2)^	0.000	0.000	0.000	0.000

^(1)^ Anova with Tukey’s post-hoc tests; ^(2)^ Independent Student t-test. ^a, b, c^ Categories with same letter do not differ significantly (*p* > 0.05).

## Data Availability

The study did not report any data.

## Supplementary Materials

The following are available online at https://www.mdpi.com/article/10.3390/nu13082648/s1, Table S1: Sample distribution according to Brazilian states and regions. Data from this study compared to data from Mapaveg; Table S2: Socio-demographic characteristics by type of diet; Table S3: Mean and standard error of WHOQOL-BREF domains adjusted by gender, age, income, education level and BMI.
