# Supplementary material for: Quality of Life of Brazilian Vegetarians Measured by the WHOQOL-BREF: Influence of Type of Diet, Motivation and Sociodemographic Data
Source: Nutrients. 2021 Jul 30;13(8):2648. doi: 10.3390/nu13082648 (PMC8400525; doi:10.3390/nu13082648)
Supplement: Supplementary file 1 [file nutrients-13-02648-s001.zip › nutrients-1291076-supplementary.pdf]

Table S1: Sample distribution according to Brazilian states and regions. Data from this study compared to data from *Mapaveg*.

	MapaVeg		Study	
**State**	**freq**	**%**	**freq**	**%**
Acre (AC)	24	0.08%	1	0.02%
Alagoas (AL)	136	0.46%	26	0.59%
Amapá (AP)	20	0.07%	3	0.07%
Amazonas (AM)	125	0.43%	22	0.50%
Bahia (BA)	731	2.50%	134	3.06%
Ceará (CE)	504	1.72%	87	1.99%
Distrito Federal (DF)	1470	5.02%	465	10,63%
Espírito Santo (ES)	364	1.24%	65	1.49%
Goiás (GO)	523	1.79%	83	1.90%
Maranhão (MA)	92	0.31%	16	0.37%
Mato Grosso (MT)	176	0.60%	34	0.78%
Mato Grosso do Sul (MS)	291	0.99%	39	0.89%
Minas Gerais (MG)	2504	8.55%	400	9.14%
Pará (PA)	205	0.70%	25	0.57%
Paraíba (PB)	291	0.99%	36	0.82%
Paraná (PR)	2267	7.74%	240	5.49%
Pernambuco (PE)	620	2,12%	100	2.29%
Piauí (PI)	104	0.36%	17	0.39%
Rio de Janeiro (RJ)	2968	10.14%	410	9.37%
Rio Grande do Norte (RN)	294	1.00%	45	1.03%
Rio Grande do Sul (RS)	2946	10.06%	332	7.59%
Rondônia (RO)	68	0.23%	7	0.16%
Roraima (RR)	1	0.00%	5	0.11%
Santa Catarina (SC)	2009	6.86%	279	6.38%
São Paulo (SP)	10365	35.40%	1469	33.58%
Sergipe (SE)	132	0.45%	30	0.69%
Tocantins (TO)	52	0.18%	5	0.11%
**TOTAL**	**29282**		**4375**	

Source: Mapaveg (MapaVeg, 2018).

Table S2: Socio-demographic characteristics by type of diet.

Characteristic	Type of diet				
	**Vegan**	**Vegetarian**	**Pesco-vegetarian**	**Semi-vegetarian**	**p***

	(n=1391)	(n=2098)	(n=330)	(n=556)	
**Gender**					
Male (n=1657)	574 (41.3%)	782 (37.3%)	79 (23.9%)	222 (39.9%)	<0.001
Female (n=2.718)	817 (58.7%)^{a}	1316 (62.7%)^{a}	251 (76.1%)^{b}	334 (60.1%)^{a}	
**Age**					
18-39 (n=2.749)	943 (67.8%)	1384 (66.0%)	156 (47.3%)	266 (47.8%)	<0.001
40 or more (n=1626)	448 (32.2%)^{a}	714 (34.0%)^{a}	174 (52.7%)^{b}	290 (52.2%)^{b}	
**Average income**					
Less than 5 minimum wages (n=1956)	620 (47.3%)	981 (49.5%)	117 (38.5%)	238 (46.4%)	0.004
Above 5 minimum wages (n=2.153)	691 (52.7%)^{a}	1000 (50.5%)^{a}	187 (61.5%)^{b}	275 (53.6%)^{a}	
**Housing location**					
Capital or metropolitan area (n=2951)	972 (69.9%)	1391 (66.3%)	228 (69.1%)	360 (64.7%)	0.064
Urban or rural area (n=1424)	419 (30.1%)^{a}	707 (33.7%)^{a}	102 (30.9%)^{a}	196 (35.3%)^{a}	
**Education: University level?**					
No (n=1633)	526 (37.8%)	851 (40.6%)	82 (24.8%)	174 (31.3%)	<0.001
Yes (n=2.742)	865 (62.2%)^{a}	1247 (59.4%)^{a,b}	248 (75.2%)^{c}	382 (68.7%)^{b,c}	
**BMI**					
Less than 25kg/m^2 (n=3077)	1068 (77.3%)	1490 (71.6%)	211 (65.1%)	308 (55.8%)	<0.001
25kg/m^2 or more (n=4338)	313 (22.7%)^{a}	591 (28.4%)^{a}	113 (34.9%)^{b}	244 (44.2%)^{c}	

* Pearson Chi-square test. Categories with same letter do not differ significantly (p>0.05).

Table S3: Mean and standard error of WHOQOL-BREF domains adjusted by gender, age, income, education level and BMI.

WHOQOL-BREF domains	Type of diet				
	Vegan (n=1391) Mean (SE) (1)	Vegetarian (n=2098) Mean (SE) (1)	Pesco-vegetarian (n=330) Mean (SE) (1)	Semi-vegetarian (n=556) Mean (SE) (1)	p(2)
**Physical health**	78.34 (0.417)^{a}	74.30 (0.339)^{b}	71.86 (0.862)^{b}	68.83 (0.665)^{c}	<0.001
**Psychological well-being**	69.83 (0.440)^{a}	66.32 (0.358)^{b}	64.47 (0.911)^{b,c}	61.96 (0.702)^{c}	<0.001

**Social relations**	64.79 (0.539)[a]	64.40 (0.438)[a]	62.28 (1.114)[a,b]	59.42 (0.859)[b]	<0.001
**Environment**	67.26 (0.393)[a]	65.81 (0.319)[b]	65.92 (0.812)[a,b]	61.87 (0.627)[c]	<0.001

(1) Estimated marginal mean (and Standard Error) adjusted by gender, age, income, education level and BMI.

(2) One-Way Ancova controlling by gender, age, income, education level and BMI followed by Bonferroni post-hoc tests. Categories with same letter do not differ significantly (p>0.05).
